# Local Variations in Current Density and Selectivity in CO_2_ Electrolyzers

**DOI:** 10.1021/acsenergylett.5c03770

**Published:** 2026-01-05

**Authors:** Pedro Arias Villaroel, Egon Kecsenovity, Csaba Janáky

**Affiliations:** † Department of Physical Chemistry and Materials Science, University of Szeged, Aradi sq. 1, Szeged, 6720, Hungary; ‡ eChemicles Zrt, Alsó Kikötő sor 11, Szeged, 6726, Hungary

## Abstract

As CO_2_ electrolysis emerges as a key technology for industrial decarbonization, scaling from lab to industrial systems introduces challenges beyond increasing the active area or the CO_2_ feed rate. Larger reactors often exhibit spatial inhomogeneities in selectivity and current density, issues overlooked in conventional performance studies. To address this, we designed, built, and tested a zero-gap flow cell for CO_2_ electrolysis that enables local monitoring of the product selectivity and current density. Gas composition is sampled at multiple points along the flow path, while current density is tracked across the cell. This approach reveals where and under what conditions parasitic hydrogen evolution occurs. In this work we present how operating parameters influence localized product formation and current density profiles in a CO_2_-to-CO electrolyzer. Our findings emphasize the importance of moving beyond averaged metrics to achieve efficient, uniform operation across the cell, an essential step for successful scale-up.

## Graphical abstract

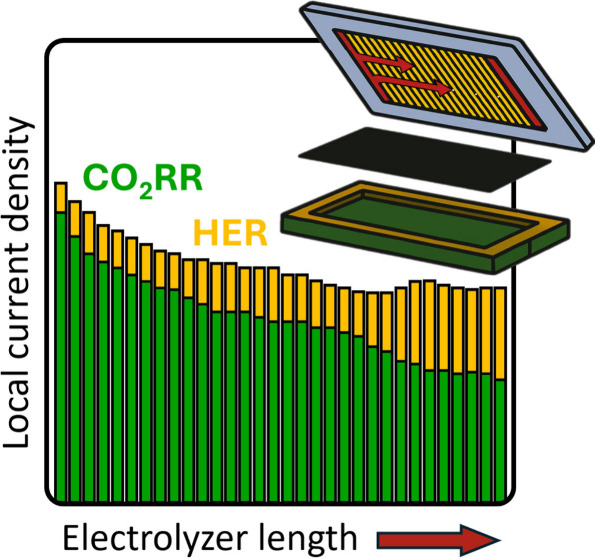

CO_2_ electrolysis offers a promising route to convert captured CO_2_ into chemical feedstocks by using green electricity. As the technology advances, industrially relevant current densities have been demonstrated, together with selective and increasingly durable electrolyzers capable of stable operation. Moreover, coupling CO_2_ electrolysis with intermittent renewable energy sources has been shown feasible, paving the way toward cost-competitive and sustainable carbon dioxide conversion.^1–4^

As this technology continues to emerge, the need arises to replicate electrolyzer performance from the lab scale to industrial-scale systems.^5,6^ This transition, however, is far from a simple matter of scaling up the active area and increasing the CO_2_ feed rate. As reactor dimensions increase, gradients in temperature, pressure, and concentration begin to play a dominant role in electrolyzer performance—also resulting in spatial inhomogeneities in current density and selectivity. These issues are negligible at the lab scale, where the cell size is small enough for parameters such as (partial) pressure, velocity, and temperature to remain essentially uniform throughout the electrolyzer.

Moreover, scaling up introduces the difficulty of relying on bulk measurements—such as overall current or outlet gas compositions at the cathode and anode—that represent only averaged values, representative for the entire active area. In reality, local variations in selectivity, current density, and ion transport may occur across the electrode area, in the form of gradients or hot-spots, yet these are masked in the collective signal recorded at the outlet.^7,8^

Such spatial phenomena have been thoroughly studied in both fuel cells and water electrolyzers.^9–13^ These devices, however, are substantially different from those of CO_2_ electrolyzers. In the H_2_ fuel cell case, two gas phase reactants are fed, and a liquid product (water) is removed.^14^ In water electrolyzers, a liquid phase reactant is fed, and two gas phase products are removed.^15^ In stark contrast, in the most advanced zero-gap CO_2_ electrolyzers, both liquid and gas feedstocks are provided; and, in the CO_2_-to-CO case discussed below, a gas phase product is formed.^16^ Having such fundamental differences means that the conclusions drawn for fuel cells and water electrolyzers are not directly applicable to CO_2_ electrolysis. Furthermore, as we move toward industrial implementation and larger cells/stack, effective management and understanding of reaction and transport conditions across the cell become crucial.

Based on the above arguments, methods for analyzing local current density and selectivity (i.e., product distribution) are now more important than ever. Conventional cell architectures provide only spatially averaged data, and dimension-dependent effects must therefore be investigated through alternative approaches. While local overpotentials, current density distributions, and selectivity gradients are often studied using Multiphysics and computational fluid dynamics (CFD) models, such models rely on assumptions that may not accurately capture real operating behavior.^17–19^

To address this challenge, we designed, built, and tested a locally resolved CO_2_RR electrolyzer cell featuring a segmented cathode current collector capable of mapping current density distribution in real time, combined with seven sampling points in the cathode chamber for online analysis of the gas products (Figure 1A,B). This setup allows simultaneous spatial and temporal monitoring of the current density and selectivity. For the sake of amplifying spatial effects, we did not introduce a gas flow field in the first iteration of the design.

**Figure 1:**
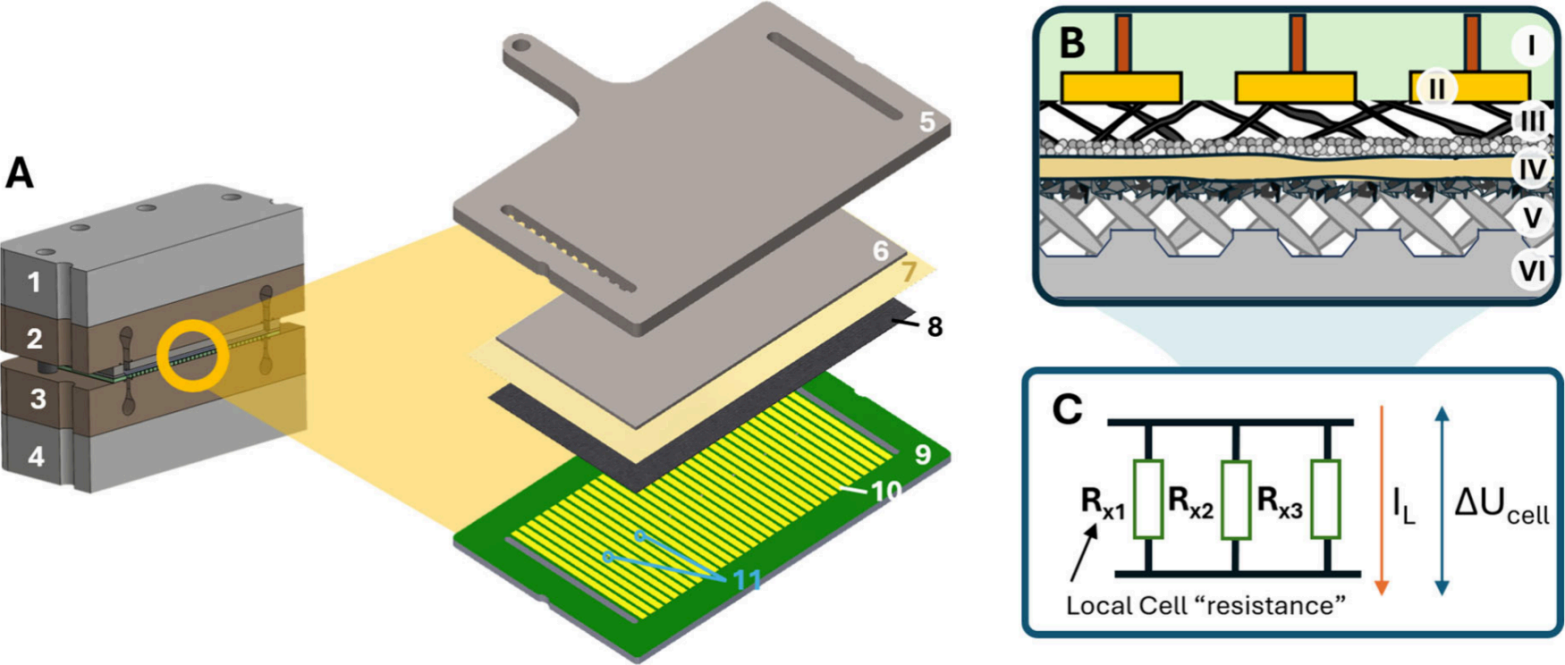
Schematic of the cell concept. (A) Cross-sectional view of the cell (left) and exploded view of the main cell components: (1) cathodic end plate, (2) printed cathodic insulator, (3) printed anodic insulator, (4) anodic end plate, (5) anode current collector made of stainless steel, (6) Ir-coated titanium porous transport electrode, PTE (7) anion exchange membrane, AEM, (8) gas diffusion electrode, GDE, (9) segmented cathode current collector, based on a printed circuit board equipped with (10) 32 individual segments, that measure the current density, and (11) 7 sampling points distributed across the length of the cell. (B) Schematics of the zero-gap cell concept: (I) Segments are built in the (II) PCB body and in contact with the (III) GDE. The (IV) AEM is pressed in between the GDE and (V) PTE, and the (VI) anodic current collector closes the cell on the other side. (C) Simplified electrical operation concept. Individual segments or areas of the cell (×1, ×2, ×3, etc.) comprise an electrical load that can be simplified as a resistance. As these change with a varying environment, the applied voltage to the whole cell will make the current distribute accordingly.

Local current density distribution and local cathode gas concentration are of particular interest in CO_2_ electrolysis for several reasons. On the one hand, there exists the side reaction of hydrogen evolution reaction (HER), which competes with CO_2_ reduction and can become dominant under certain conditions, such as an improper management of electrolyte content in the gas diffusion electrode (GDE).^20^ This process not only enhances mass transport limitations but also increases the apparent cathodic activation resistance toward CO_2_ reduction, as the partial current density toward C-based products decreases, and the reaction pathway shifts towards the HER.^17^ On the other hand, unlike in water electrolysis, CO_2_ is directly gas-fed into the cell, so its partial pressure, a key driver of its accessibility to active sites, gradually decreases from inlet to outlet due to product formation, carbonate ion migration through the anion exchange membrane (AEM), and an overall pressure drop. This limits the availability of the reactant from the bulk-phase to the catalyst layer, thus impacting the mass transport resistance.

Due to the presence of gradients in electrolyte content, reactant partial pressure, and temperature, nonuniform local operating conditions develop across the electrolyzer. As a result, local variations arise in mass transport and apparent activation resistances, as well as in membrane resistance, which depends on the dominant charge carrier (CO_3_^2–^, HCO_3_^–^, or OH^–^).^21–23^ These variations can be phenomenologically represented through an equivalent electrical circuit in which each local region contributes a distinct resistance corresponding to the local electrochemical and transport phenomena (Figure 1C). From a physical perspective, the electrolyzer operates under a globally applied potential; that is, all points in the cell share the same voltage between equipotential current collectors. The nonuniformity of local conditions therefore results in spatial variations in current density rather than in potential. In a steady-state operation, these variations in current density reflect the gradual change in local resistances along the cell.^24,25^

The custom-designed cell is a 50 cm^2^ zero-gap CO_2_ electrolyzer equipped with a cathode current collector fabricated as a printed circuit board. This collector contains 32 segments, each measuring the local current density independently at a given cell voltage that is applied to the whole cell equally. From an electrical point of view, cathode and anode are connected to the power source, just as in any other conventional electrolyzer cell. The voltage is applied to the cell, and current density is measured individually using a shunt resistor for each segment (calibration of the shunts is included as Figure S1, with a brief explanation of the working principle). The linear arrangement of these segments, positioned sequentially from the inlet to the outlet, enables operando, in-line mapping of the current density distribution across the cell. The effective active area of each of these segments is 1.56 cm^2^ each, and their reading velocity is below 0.5 s for all 32 pads, allowing one to track very fast changes in the current density profile, very quickly, and in a very tight space. A view of the custom-designed current collector, including the associated circuitry, is included in Figure S2.

The cathode current collector includes seven intermediate sampling points, evenly spaced, and located in the middle of the electrolyzer, connected to a mass spectrometer for online measurement of the local cathode gas composition. The instrument withdraws a negligible gas volume from the electrolyzer (approximately 5 mL min^–1^), representing less than 1% of the CO_2_ feed rate. Gas sampling is managed through a 3D-printed component integrated into the cell equipped with printed capillary conduits. This printed part also accommodates the cathode inlet and outlet connections and functions as an electrical insulator for the cell. Figure 1A shows the cell assembly and its assembly principle, while Figure S3 provides a complete view of the assembled cell.

The cell was tested for CO_2_ electrolysis, specifically for the conversion of CO_2_ to CO. In all experiments, the cathode consisted of silver nanoparticles spray-coated onto a Sigracet 39BB gas diffusion layer (GDL) and operated in a zero-gap configuration (Figure 1B). A PiperION (40 μm) membrane was used as the separator. The anode catalyst, Ir-black, was spray-coated onto a porous titanium layer (titanium frit). During the operation, CO_2_ was supplied from a heated source maintained at 60 °C. The anolyte, a 0.05 M CsHCO_3_ solution, was continuously recirculated through the anode compartment at a flow rate of 20 mL min^–1^ cm^–2^ and 60 °C. The cell was operated galvanostatically at various current densities (200, 300, 400 mA cm^–2^). The cathode gas outlet was analyzed continuously using an online process gas analyzer, periodically calibrated with predefined CO_2_, CO, and H_2_ gas mixtures (). All further experimental details are provided in the Supporting Information.

First, the effect of the employed current density on the cell performance was investigated. The applied current density was increased sequentially from 200 mA cm^–2^ to 300 mA cm^–2^ and then to 400 mA cm^–2^ (Figure 2B). The inlet CO_2_ flow rate was maintained at 12 mL min^–1^ cm^–2^ throughout these experiments. Cell voltage and selectivity results are shown in Figure 2A and C, respectively. The cell voltage values are within the range typically observed for such zero-gap electrolyzers at these current densities,^26–28^ while the CO-selectivity is exceptionally high (note that only trace amounts of H_2_ were detected), which results in CO/H_2_ ratio of over 200! Figure 2D, E, and F present the current density distribution profiles at each operating condition; this is 200, 300, and 400 mA cm^–2^, respectively. In all cases, CO_2_ entered the cell from the left and passed through the active area—perpendicular to the pads—exiting at Pad 32. Figure 2D indicates that even at relatively low current density, significant inhomogeneities in the current distribution are present. The standard deviation from the intended set current density (200 mA cm^–2^) is 29 mA cm^–2^. The absolute value of this deviation increases with higher current densities, reaching 47 mA cm^–2^ at 300 mA cm^–2^ (Figure 2E) and 55 mA cm^–2^ at 400 mA cm^–2^ (Figure 2F). This deviation becomes more pronounced toward the outlet region (Pad 22 onward), resulting in a marked decrease in local current density. Interestingly, despite these variations in the current distribution, the average selectivity toward CO remained high, as mentioned before.

**Figure 2:**
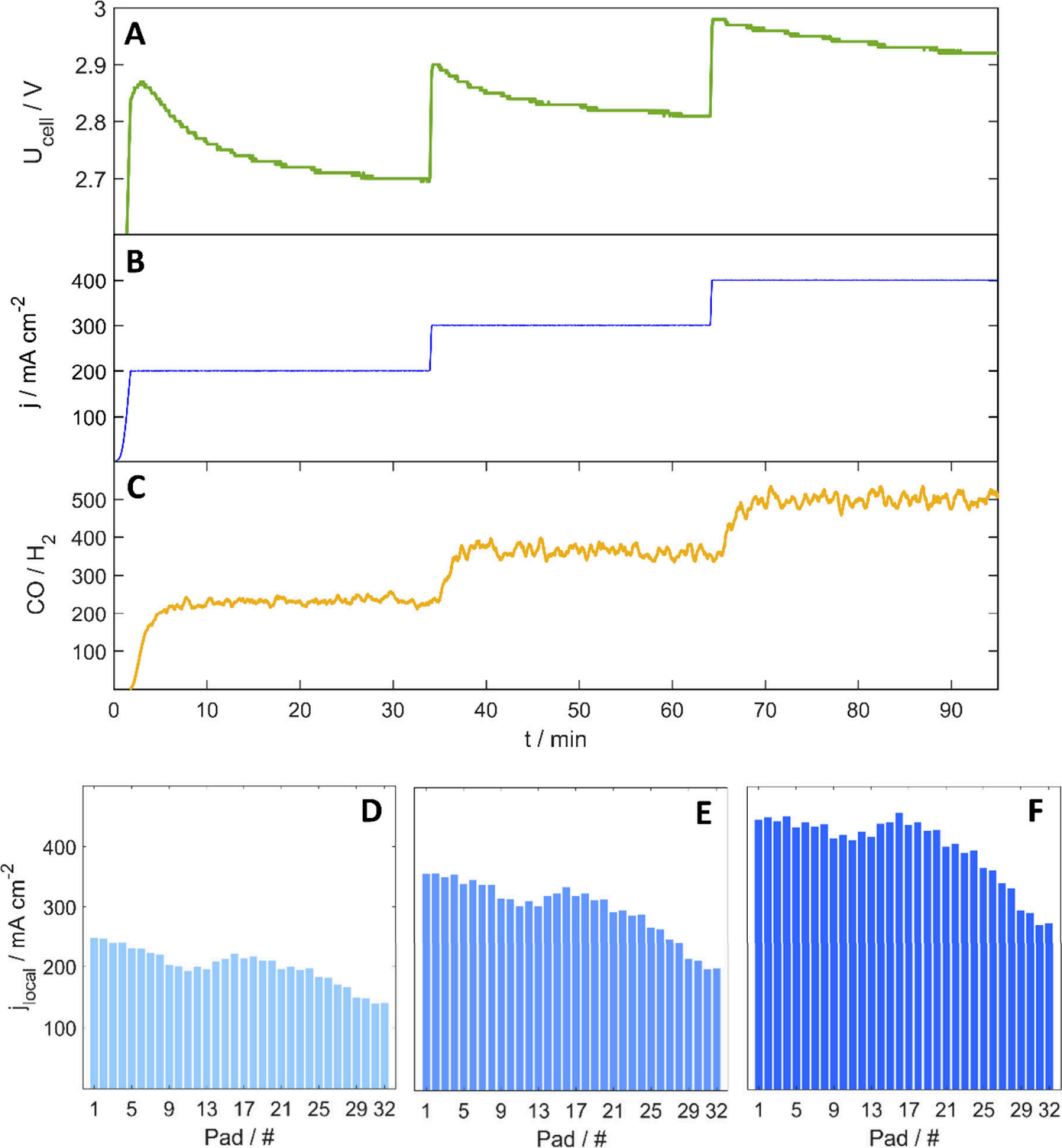
Effect of current density on the (A) cell voltage, (B) current density intervals, and (C) product selectivity. Panels (D), (E), and (F) show the corresponding current density profiles at 200, 300, and 400 mA cm^–2^, respectively, taken 15 min after each current interval start. The CO_2_ inlet is located at pad number 1, and the outlet is located at pad number 32.

Areas of higher current density in the electrolyzer indicate a consistent variation in the cell’s overpotential resistances. Factors such as mass transport limitations, activation overpotentials, membrane resistance, geometric deviations, or a combination of these contribute to an increased resistance to current flow in the latter part of the cell, resulting in a lower local current density. In addition, we examined the influence of anolyte flow direction by reversing it after 1 h of operation (400 mA cm^–2^) and analyzing the resulting current density profiles. A consistent downward trend from the cathode gas inlet to the outlet was observed under both operating modes. The corresponding profiles (Figure S5), followed by a more detailed description of the methodology, are provided in the Supporting Information.

In-situ experimental measurement of current density decrease along the channel has not been reported previously in the literature, but its occurrence has been analyzed through Multiphysics models. Several authors have modeled the changes in local current density along the channel of electrolyzers in gas diffusion electrodes, for CO_2_ to CO conversion, showing similar downward curves to those presented in this work.^29,30^ These works suggest a sharp decline in local current density in the initial length of the electrolyzer, although the specific profile decrease varies greatly with different potentials and catalyst layer characteristics.

Subsequently, we performed an online, operando analysis of the concentration of products in the cathode compartment, for the same current densities. For this purpose, the sampling points, which are distributed across the length of the electrolyzer from inlet to outlet, are in the middle of the width. The extracted cathode gas amount remained always below 1% of the nominal CO_2_ electrolyzer feed, to avoid disturbing the electrochemical performance. The sampled gas is passed through a condenser to remove water and then analyzed by using an online mass spectrometer. Further details on the sampling methodology are covered in the SI, together with a simplified system diagram (Figure S6).

Figure 3 illustrates the spatial evolution of product concentrations across the electrolyzer, as measured at the sampling points and at the outlet, for three different current densities once steady-state is reached. In all cases, a progressive consumption of CO_2_ is observed along the reactor pathway, accompanied by the formation of CO. Naturally, this trend becomes more pronounced at higher current densities, with local CO_2_ depletion taking place. Specifically, at 300 mA cm^–2^, CO_2_ is nearly exhausted by the outlet sampling points, while at 400 mA cm^–2^, depletion occurs around sampling point 6. Note again that no flow pattern was employed in this case. As shown in Figure 3B,C, the low CO_2_ concentrations in the latter stages of the reactor lead to a shift in selectivity toward the HER. Notably, there is a mismatch between the concentration of products from the sampling points (even the last one before the exit) and the averaged outlet. This mismatch, which is discussed more in depth in what follows, suggests nonuniform reactant distribution within the cathode compartment.

**Figure 3:**
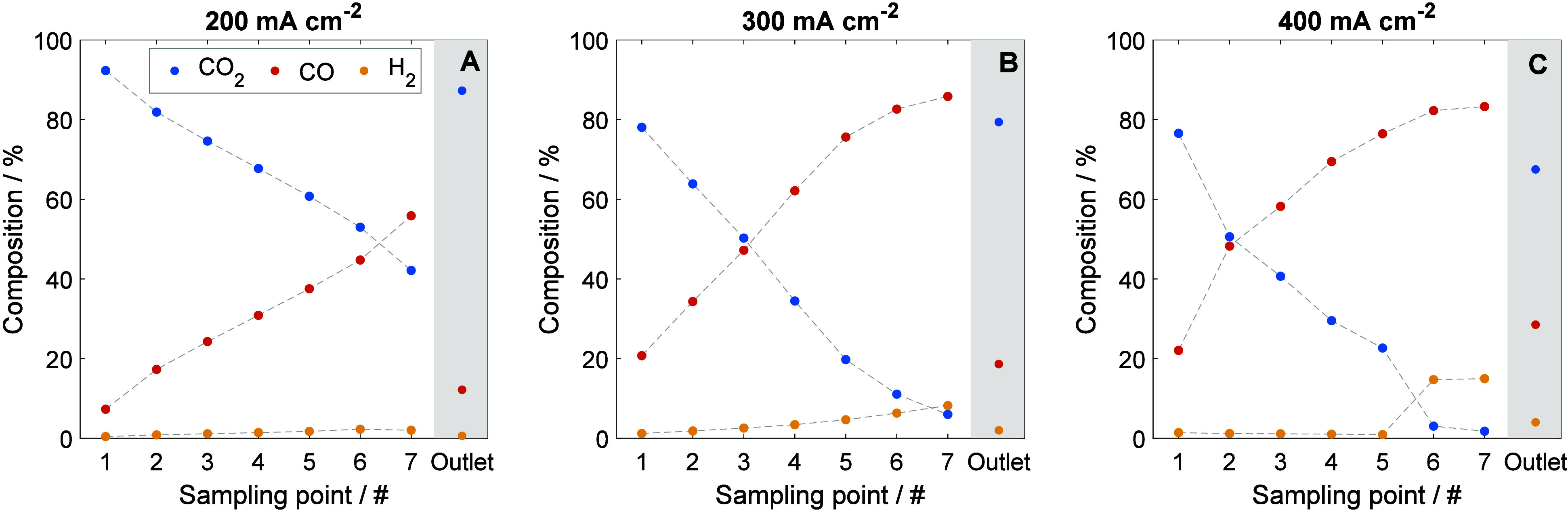
Concentration profile at (A) 200 mA cm^–2^, (B) 300 mA cm^–2^, and (C) 400 mA cm^–2^, together with the averaged outlet. These were taken 15 min after each step, in the span of ∼30 min, once steady-state was reached at 2.75, 2.87, and 3.00 V.

To further analyze the effect of CO_2_ starvation on the current density profile, the CO_2_ feed rate was varied. Starting from an inlet flow rate of 12 mL min^–1^ cm^–2^, which yielded high selectivity toward CO and a steady-state cell voltage of 2.92 V, the CO_2_ feed was decreased stepwise to 10, 8, 6, and 4 mL min^–1^ cm^–2^. At the lowest flow rate, achieving 100% faradaic efficiency (FE) for CO is stoichiometrically impossible; this experimental design was intended specifically to study the effects of the availability of CO_2_ on hydrogen evolution. As the CO_2_ flow rate decreased, selectivity toward CO dropped sharply initially and then stabilized after a few minutes, while parasitic HER became more pronounced (Figure 4C). This effect was most significant at the lowest flow rates (6 and 4 mL min^–1^ cm^–2^), where the CO/H_2_ ratio fell below 3.

**Figure 4:**
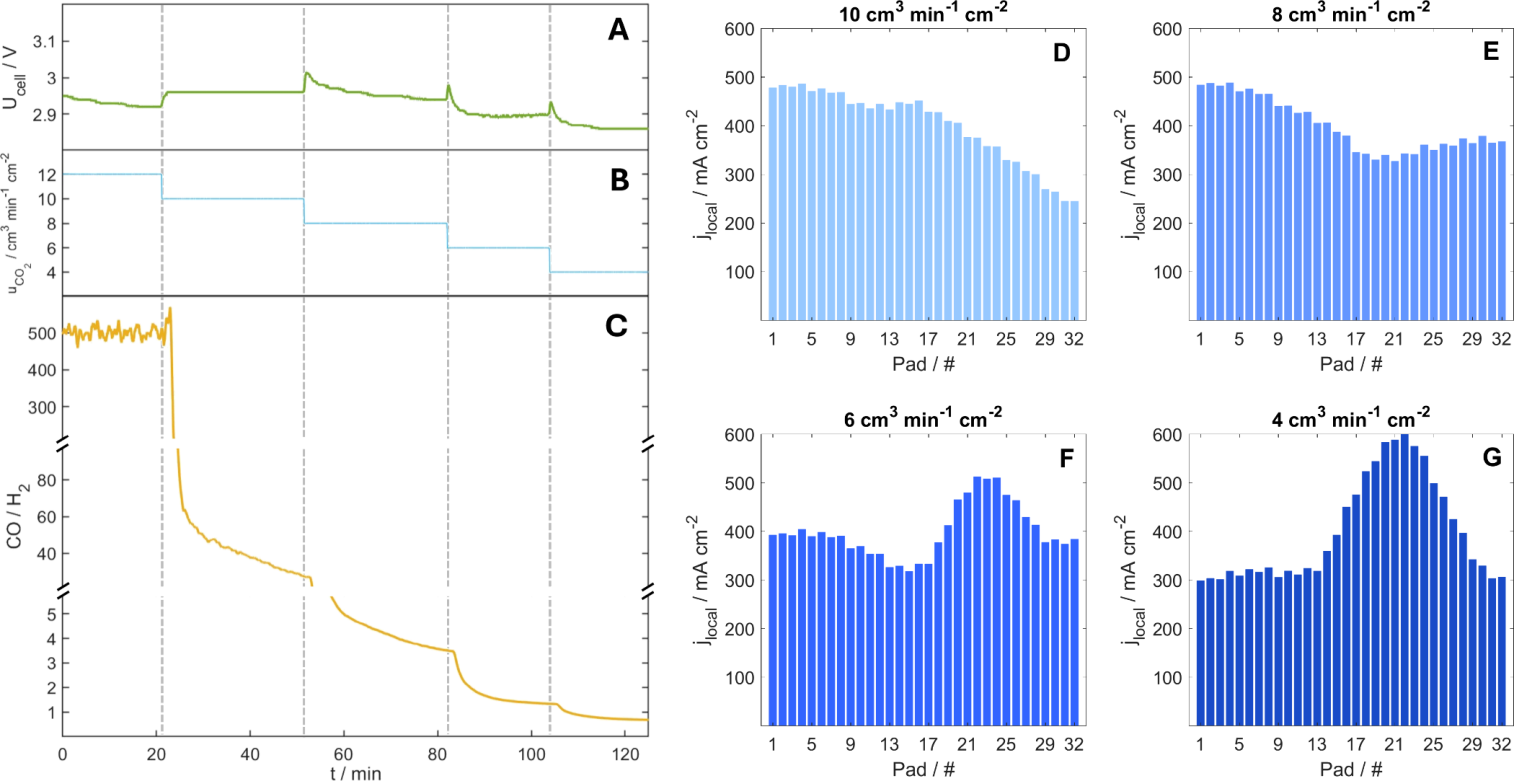
Effect of CO_2_ feed flow rate (B) on (A) cell voltage and (C) selectivity. Panels D–G show the corresponding current density profiles at normal 10, 8, 6, and 4 cm^3^ min^–1^ cm^–2^, respectively, taken 15 min after each flow rate interval start. The electrolyzer current density during the entire period was 400 mA cm^–2^. The vertical dashed lines represent the change in flow rate.

Corresponding trends were also observed in the spatially resolved current density profiles. Figure 4D–G shows the stabilized current density distributions, recorded 15 min after each flow rate adjustment, for 10, 8, 6, and 4 mL min^–1^ cm^–2^ intervals. In Figure 4E, the last one-third of the electrolyzer, which previously exhibited sluggish activity, begins to recover as the CO selectivity decreases. In Figure 4F, a pronounced current density peak develops in the third part of the electrolyzer, the previous most inactive region, which becomes even more prominent when the CO_2_ feed is further reduced to 4 mL min^–1^ cm^–2^ (Figure 4G). This result highlights the importance of an adequate CO_2_ feed in electrolyzers, not only to maintain high selectivity, but also to ensure a uniform current distribution across the cell. It must be noted that with changing the CO_2_ feed rate, the CO_2_ pressure also changes within the cathode compartment.

The local current density acquisition software refreshes the complete current density profile every 0.33 s. This high resolution enables very fast observation of changes in the current distribution, allowing for the accurate tracking of dynamic trends within the electrolyzer. Throughout these experiments, the evolution of the current density profile could be monitored in real time, providing direct insight into how long the electrolyzer took to respond to variations in the CO_2_ feed flow rate change. As shown in Figure 4, the system’s response to feed changes is not instantaneous; rather, both the cell voltage (Figure 4A) and CO selectivity (Figure 4C) require a few minutes to reach a new steady state. Correspondingly, the local current density distribution undergoes a similar transition before stabilizing into a new steady-state profile, over several minutes, when changing the CO_2_ flow rate from 8 to 6 normal cm^3^ min^–1^ cm^–2^. In Figures S7–S9, we recorded the transition phase of the current density profile for 10 to 8, 8 to 6 and 6 to 4 normal cm^3^ min^–1^ cm^–2^. These transitions to changes in operational parameters are also included in the Supporting Information, as a short video covering the changes throughout the measurement in Figure 4.

The underlying cause of this delayed stabilization is complex and convoluted. We hypothesize that a variation in the local current density implies a change in the summed local resistance, which is determined by the cathodic and anodic mass transport resistances, activation overpotentials, and membrane resistance. Given that the change in CO_2_ feed flow rate is confined to the cathode, changes in anodic resistance could arguably be neglected. Therefore, the observed evolution in the current density profile likely arises from a combination of variations in the cathodic mass transport resistance, local activation energy barriers, and membrane resistance.

Figure 5 presents the concentration profiles of products under the conditions of CO_2_ starvation, evaluated at a nominal current density of 400 mA cm^–2^. Three CO_2_ feed scenarios were investigated, corresponding to CO_2_ flow rates of 8, 6, and 4 normal cm^3^ min^–1^ cm^–2^. The associated current density distributions, which exhibit trends consistent with those shown in Figure 4E–G, are provided in Figure S9. These results are consistent with the behavior previously observed in Figure 3: as local CO_2_ availability decreases, the system increasingly favors hydrogen evolution, resulting in H_2_ becoming the predominant product. In addition, as the CO_2_ feed rate is reduced, the onset of hydrogen is displaced to earlier sampling points. This shift is more pronounced at lower CO_2_ feed rates and appears to influence the current density profile, including the emergence of localized peaks (see Figure S10). These findings are in accordance with previous reports of CO_2_ electrolysis mapping,^31^ but the emergence of current density spikes due to the lower thermodynamic potential of hydrogen is a new observation here.

**Figure 5:**
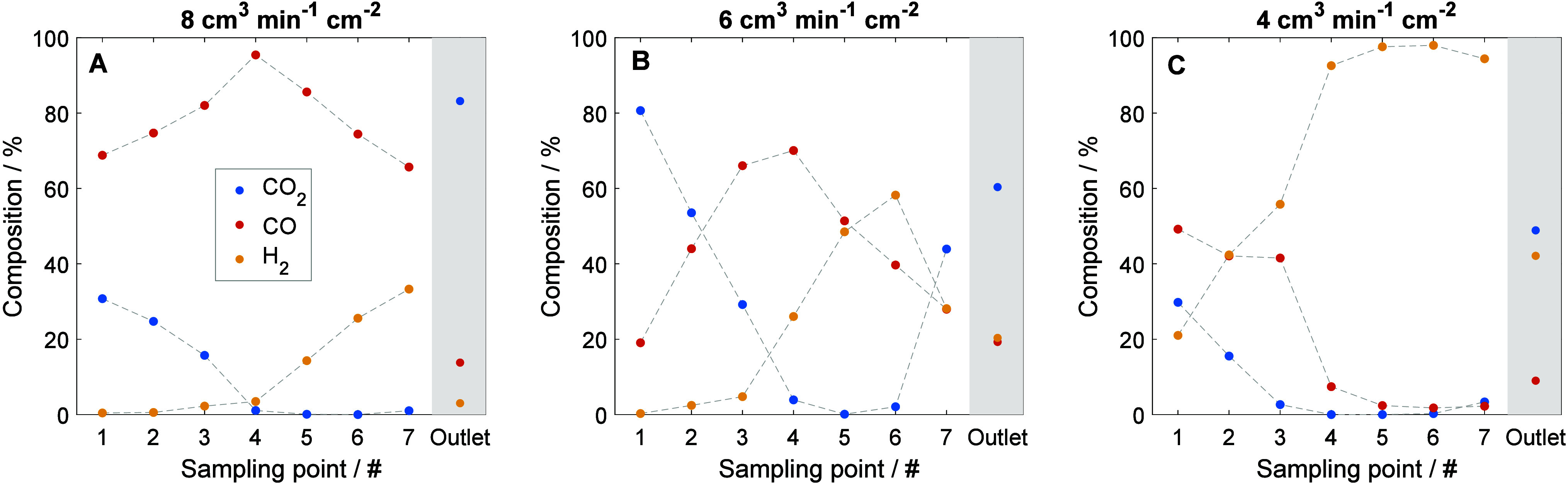
Concentration profile at normal (A) 8 cm^3^ min^–1^ cm^–2^, (B) 6 cm^3^ min^–1^ cm^–2^, and (C) 4 cm^3^ min^–1^ cm^–2^, together with the averaged outlet. These were taken 15 min after each step in the span of ∼30 min. Cell was operated at 400 mA cm^–2^.

The concentration profile corresponding to a CO_2_ feed rate of 4 cm^3^ min^–1^ cm^–2^ is particularly noteworthy. From approximately sampling point 4 (located in the center of the active area) onward, H_2_ becomes the dominant gas species, accounting for over 90% of the detected products. This observation suggests local CO_2_ depletion in the latter stages of the reactor under these operating conditions. Of particular interest is the potential relationship between this phenomenon and the local distribution of the electrolyte within the gas diffusion layer, which may play a critical role in modulating product selectivity and current density locally. An interesting approach in this sense would be to analyze the product distribution with the local relative humidity.^32^

As shown in Figure 3, there was a mismatch between the concentrations measured at the interim sampling locations and the outlet of the electrolyzer. Although the trends—CO formation, CO_2_ consumption, and H_2_ production—were consistent, the concentrations at the sampling points were significantly higher. This suggested that CO_2_ was not evenly distributed across the cell’s transversal axis, likely due to poor flow distribution. To address this, we tested a modified cathode current collector featuring a serpentine flow field to improve the reactant distribution. Figure 6 compares results obtained by using two different cathode flow field configurations. Both analyses were carried out under identical conditions, namely, a nominal current density of 400 mA cm^–2^, an operation temperature of 60 °C, and a dry CO_2_ feed of 12 normal cm^3^ min^–1^ cm^–2^. In Figure 6A, the flat cathode current collector is used (Figure 1A, component 9), as in all measurements presented so far, as a benchmark. In Figure 6B, this component is modified with a serpentine flow field (technical drawing in Figure S11), which is generally employed in different gas-fed electrolyzers.

**Figure 6:**
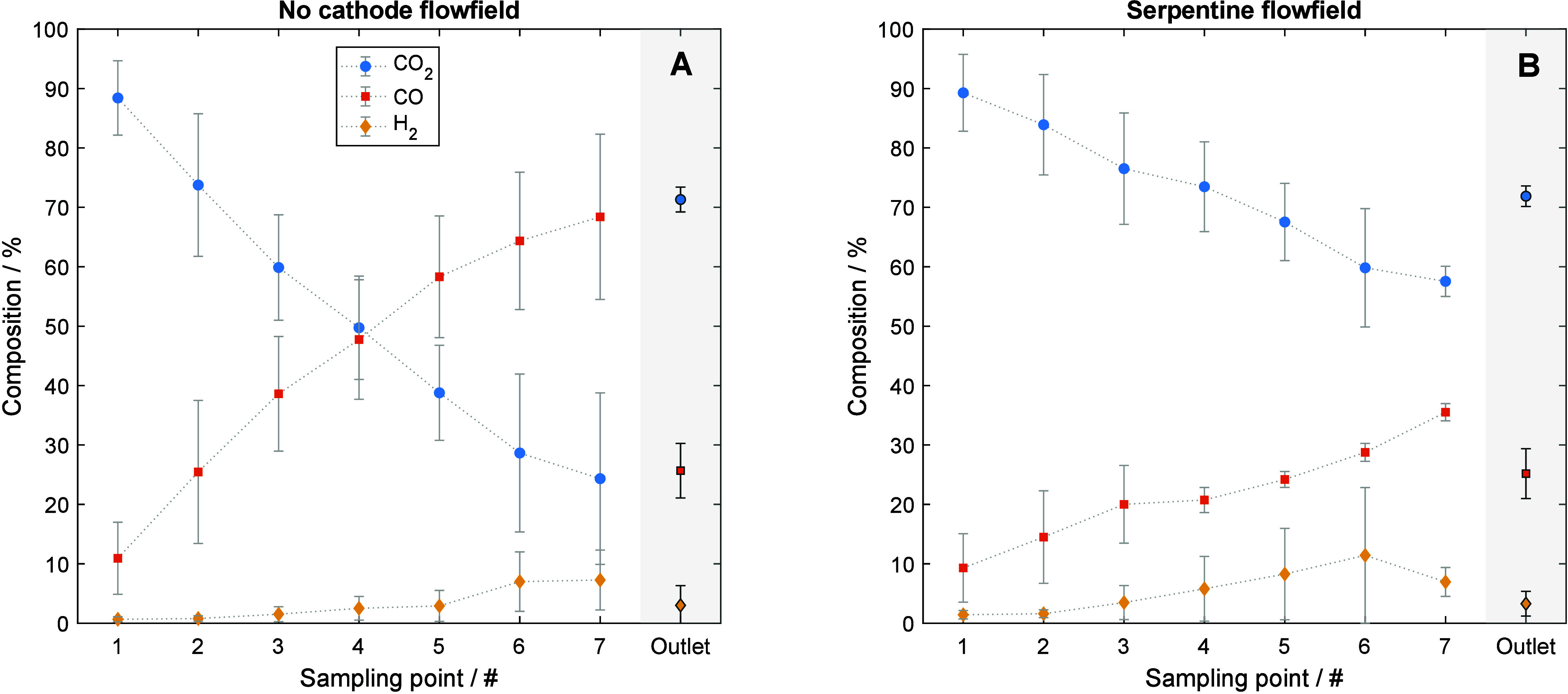
Cathode gas online analysis. (A) Flat cathode current collector; gas is forced through the GDE. (B) Modified collector with serpentine flow field, design drawing in Figure S11. All online measurements were taken at 400 mA cm^–2^, 12 normal cm^3^ min^–1^ cm^–2^. Error bars represent the standard deviation from at least three independent measurements.

For both configurations, we observe a clear concentration gradient along the sampling channel, consistent with plug-flow reactor behavior, in which CO_2_ is progressively consumed and converted to CO and a small amount of H_2_ as the gas moves downstream, which is consistent with the concentration profiles shown before. The H_2_ fraction remains low across all sampling points but increases slightly toward the outlet. When no flow field is employed, product concentrations measured at individual sampling points inside the cell are substantially higher than those found at the outlet (right part of Figure 6A). This indicates a nonuniform gas distribution within the cell, having regions where CO_2_ can pass without reaction. Such heterogeneity suggests uneven reactant access and limited mixing, leading to spatial variations in the conversion and selectivity. More specifically, we hypothesize that there is a substantial amount of the CO_2_ that bypasses the active area, through the areas of least resistance in the gas diffusion electrode. The reasons for these areas of less gas-flow resistance in the electrode are not clear, but they can range from defects on the homogeneity of the electrode void channels, wide tolerances in the current collectors and/or uneven compression pressure being applied on the electrode or, more interestingly, the disruption brought by a saturation of electrolyte content in the cathode compartment (or “flooding”).^33,34^

In order to rule out the influence of the cell architecture on the cathode gas (possible) improper distribution, we simulated the CO_2_ feed flow through the external cell port and GDE. This way, we demonstrated that the reported results are not a product of the cell architecture. The results of these, along with the experimental details, are collected in Figures S13 and S14, along with a technical drawing specifying the design of the cathode insulator plate (Figure S12).

We hypothesize that wetted regions in the GDE restrict CO_2_ gas transport by reducing the effective gas diffusion pathways, even if they do not completely block its flow. Beyond this, persistent flooding can create isolated liquid pockets where local concentrations change (e.g., the concentration of diluted CO_2_ decreases) due to limited convective exchange. Such conditions can lead to shifts in local selectivity and modifications of the gas flow pattern by water physically blocking the previously available pathways.^35^

Consequently, measurements taken within the channel provide a more accurate picture of local reaction efficiency and concentration, while outlet sampling captures a mixed, averaged composition. These findings emphasize the importance of reactor design and the need of applying a flow field to achieve uniform gas distribution all over the cell.

Introducing a serpentine flow field (Figure 6B)—a configuration widely used in fuel cells,^36,37^ water electrolyzers,^38,39^ and increasingly in CO_2_ electrolysis^40^—substantially improves gas distribution.

Variations in current density and selectivity across the *X*–*Y* plane can lead to transversal gradients in species concentration. We hypothesize that the serpentine channels primarily serve to collect electrolyte^41^ and remix species concentrations during electrochemical operation, promoting a more uniform gas distribution, consistent with the results shown in Figure 6.

The serpentine channel then promotes uniform CO_2_ transport and minimizes spatial concentration gradients, particularly in the transversal axis, resulting in product compositions at internal sampling points that closely match the outlet. It is also clear that the flow-field is suboptimal since locally much higher conversion rates can be achieved without this flow field (Figure 6A).

## Concluding Remarks and Future Steps

We demonstrated the inhomogeneity of current density distribution in a custom designed CO_2_ electrolyzer cell and its variations with respect to nominally applied current density and reactant starvation. We observed a consistent trend in product formation along the length of the electrolyzer, and the tendency is toward CO_2_ starvation and product accumulation. We have shown that upon CO_2_ starvation, there is a rapid growth in the hydrogen concentration in subsequent sampling locations. Interestingly, this is coupled with a spike in the current density signals in the area, suggesting a link between CO_2_ depletion, selectivity decrease, and current density changes, all within a local area of a larger electrolyzer cell. We have also explored that the system is highly dynamic, continuously changing local current densities. In addition, several minutes are necessary to achieve stable current density profiles when there is a change in operational parameters such as the CO_2_ feed flow rate.

When comparing the interim values with the outlet, averaged concentration, it becomes evident that the flow distribution of the cathode gas is not homogeneous, leading to dead volumes or bypasses, which in turn reduce the overall yield of the electrolyzer cell. We hypothesize that the inhomogeneities observed in the concentration profile are not due to pure flow limitations but are electrochemical in nature. This is discussed below.

Water accumulation in the GDE limits the transport of CO_2_ by forcing access primarily through diffusion. Reduced CO_2_ availability can shift the selectivity toward hydrogen, consistent with the trends shown in Figure 5. Additionally, blocked diffusion pathways alter the cathode gas flow, generating additional inhomogeneities in concentration.

Several authors have demonstrated water accumulation and salt deposition for zero-gap CO_2_ electrolyzers.^42,43^ But their dynamic occurrence within the whole GDE area has been scarcely reported. In this direction, operando synchrotron radiation imaging of the electrolyte and gas distribution demonstrated the heterogeneous accumulation of crystals, electrolyte, and bubbles through the entire GDE.^44^

Looking ahead, critical questions still need to be addressed. Variations in local resistances and water accumulation across the entire active area should be systematically investigated to better understand concentration and current density gradients, ultimately enabling the more efficient operation of CO_2_ electrolyzers. Further research should focus on elucidating the mechanisms of cathode gas transport, examining how these pathways are influenced by electrochemical factors, and determining their impact on local current density and product selectivity. Integrating advanced diagnostic tools like the one presented here, and modeling approaches could provide deeper insights into these phenomena, paving the way for improved cell designs and operational strategies.

## Supplementary Material





## Notes

Supporting video (MP4)
Additional information on the electrolyzer cell and additional electrochemical data (PDF)
