# Supplementary material for: Local Variations in Current Density and Selectivity in CO2 Electrolyzers
Source: ACS Energy Lett. 2026 Jan 5;11(2):2029–37. doi: 10.1021/acsenergylett.5c03770 (PMC12910950; doi:10.1021/acsenergylett.5c03770)
Supplement: Supplementary file 2 [file nz5c03770_si_002.pdf]

– Supporting Information –

Local Variations in Current Density and Selectivity in CO_2 Electrolyzers

Pedro Arias Villarroel,[1,2] Egon Kecsenovity,[2] Csaba Janáky[1,2,]*

[1]Department of Physical Chemistry and Materials Science, University of Szeged, Aradi sq. 1, Szeged, 6720, Hungary

[2]eChemicles Zrt, Alsó Kikötő sor 11, Szeged, 6726, Hungary

AUTHOR INFORMATION

Corresponding Author

*Csaba Janáky, janaky@chem.u-szeged.hu

Experimental methods and materials

Experimental Materials

$CsOH \cdot H_2O$ (99.95%) and silver nanopowder (average particle size < 100 nm, 99.5% purity, 5.0 $m^2 \cdot g^{-1}$) were obtained from Sigma-Aldrich. The PiperION membrane (40 μm thick, PiperION-A40-HCO_3) and the PiperION ionomer dispersion (PiperION-A5-HCO_3-EtOH, 5 wt% in ethanol) were supplied by Versogen. The Ir-black catalyst and the Sigracet 39 BB carbon paper was purchased from FuelCellStore. Ultrapure deionized water (resistivity = 18.2 MΩ·cm) was produced using a Millipore Direct-Q 3 UV system and used for all solution preparations. The $CsHCO_3$ electrolyte was prepared by bubbling CO_2 gas through a CsOH solution until saturation (at least 30 minutes).

Electrode Preparation

The cathode catalyst ink was prepared by dispersing silver nanopowder with 5 wt% PiperION ionomer ($m_{(ionomer)}/(m_{(ionomer)} + m_{(Ag)})$) in a 1:1 isopropanol/water solvent mixture to obtain a silver concentration of 24 $mg \cdot cm^{-3}$. The anode ink consisted of Ir-black nanoparticles with 15 wt% PiperION ionomer ($m_{(ionomer)}/(m_{(ionomer)} + m_{(IrOx)})$) dispersed in the same solvent at a concentration of 18 $mg \cdot cm^{-3}$ (Ir). The Ir-dispersion was homogenized by magnetic stirring at 600 rpm, while the silver dispersion was prepared using a high-power sonotrode for 3 minutes, followed by 20 minutes of ultrasonication in a water bath maintaining the bath temperature below 35 ˚C. During spray coating, the dispersion was continuously magnetically stirred

Gas diffusion electrodes (GDEs) were fabricated with ultrasonic spray coating. The Ag dispersion was spray-coated onto preheated Sigracet 39BB on a 100 ˚C hotplate until achieving a catalyst loading of 1.0 ± 0.1 $mg \cdot cm^{-2}$ (Ag). The Ir-dispersion was applied similarly onto a

porous Ti layer to reach 1.0 ± 0.1 mg·cm^{-2} (Ir). Loadings were determined by weighting uncoated and coated supports, before and after deposition, respectively.

Membrane Pretreatment

Before assembly, the membranes were ion-exchanged for at least 24 hours in 1 M CsOH, replacing the solution after the first 5 hours. Before assembly, the membrane is rinsed in deionized water.

Current density reading principle

The current density measurement is based on the voltage drop across shunt resistors. Each segment of the cathode current collector is connected in series with a low-resistance shunt. When current flows through a segment, it generates a small voltage drop across the resistor, proportional to the current. This voltage is measured, amplified, and digitized using a custom electronic design. A microcontroller processes the signal and sends it to a terminal, where the current density profile is displayed in real time and logged, through a custom developed MATLAB software. The shunt resistors calibration is shown below.

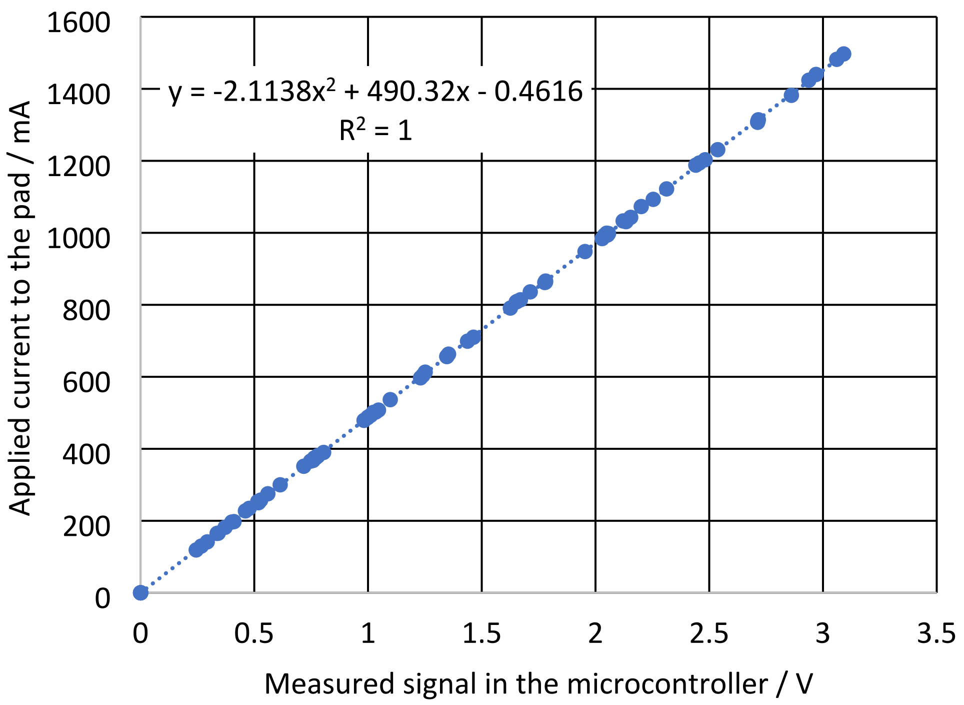

Figure S1. Recorded response in the microcontroller to applied current on the individual pads of the custom-made cathode current collector.

The cathode current collector is a gold coated printed circuit board. The current is transferred collectively from the collector's attachment to the pontentiostat and to the 32 segments using 16 (0.35 mm) filled and capped vias per pad to ensure the thin gold surface does not exhibit potential gradients in any direction. This feature was thoroughly tested before proceeding with the electrochemical experiments.

The current sensing segments are 2 mm thick and spaced 1 mm apart. They have negligible height in the Z-direction (5 µm), meaning they do not protrude above the PCB surface. Sampling points have a diameter of 0.8 mm and are located 12 mm apart. This design prevents interference with the overall compression of the GDE and adheres to a conventional approach. In order to provide a clearer view of the printed circuit board, below we depict a top and bottom view of the complete circuitry of the component.

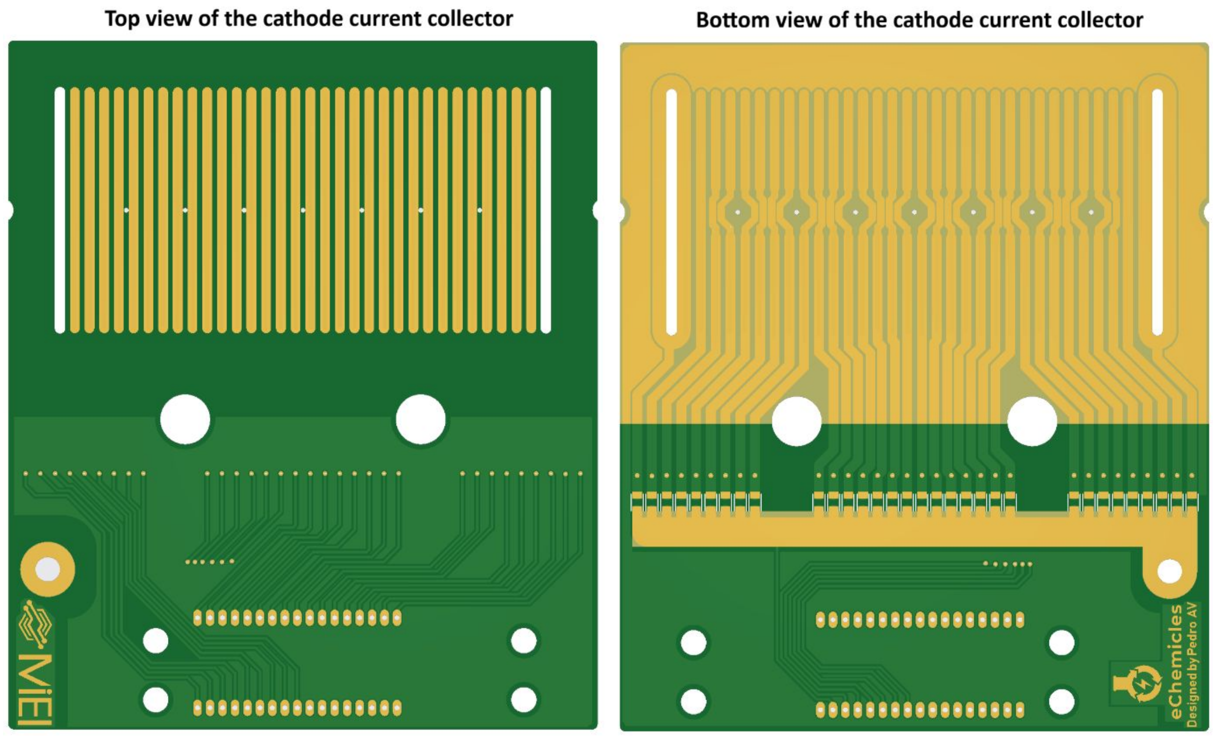

Figure S2. Top and bottom view of the cathode current collector. The GDE is positioned on the pads shown on the upper part of the figure on the left, when the cell is assembled.

Electrolyzer cell and test station

The custom-designed zero-gap CO_2 electrolyzer used in these measurements consists of several key components. The outer structure includes cathode and anode endplates, made of FR4 and shown in grey in **Figure S3**. Brown resin-printed insulator plates are positioned between the endplates; these contain the fluid inlets and outlets for both electrodes, as well as a capillary conduit for online analysis on the cathode side. The sampling streams are recovered from the top endplate.

Inside the enclosure, the anode current collector is made of 316L stainless steel, while the segmented cathode current collector is gold-coated on a printed circuit board, as described earlier. PTFE gaskets are used to hold the electrodes and membrane in place and ensure sealing.

Additional sealing is provided by o-rings, which fit into grooves engraved into the relevant components. The cell assembly (without screws) is shown below.

The assembly of the cell is performed from bottom to top as in **Figure S3**. Insulator plates are equipped with alignment pins that ensure their adequate positioning with respect to the current collectors. The tightening of the cell is carried out in a star-like fashion, increasing the applied torque sequentially: 1, 3, 6 N m.

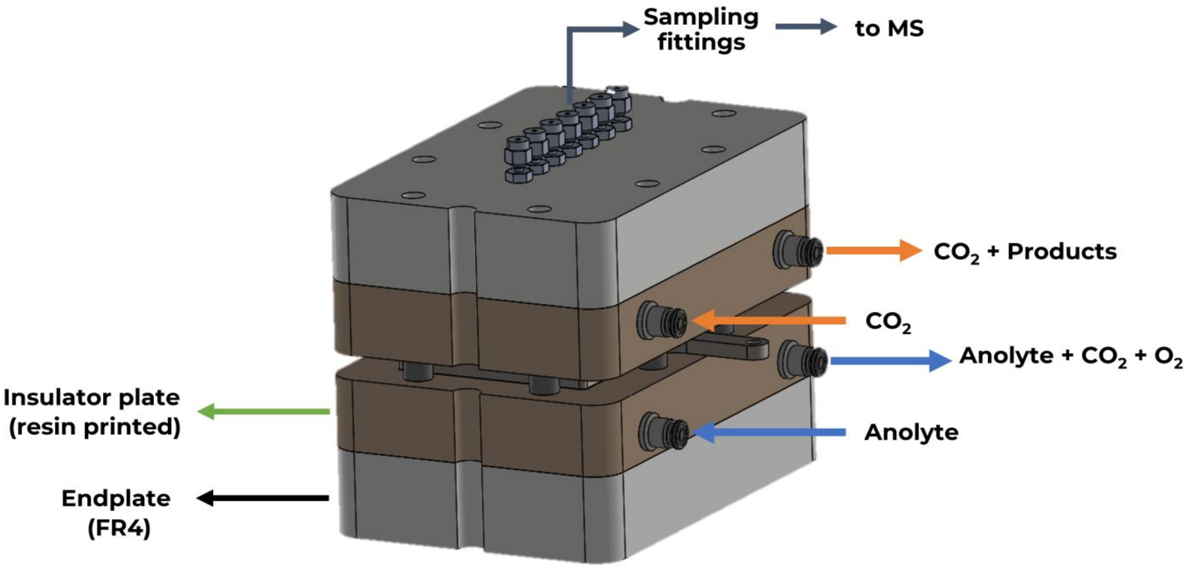

Figure S3. Schematic representation of the electrochemical cell assembly used for CO_2 electrolysis experiments.

Gas samples from the seven points are taken sequentially, starting from the outlet and moving toward the inlet. The amount of cathode gas extracted is always below 1%% of the nominal CO_2 feed to avoid disturbing electrochemical performance. Each sample passes through a condenser to remove water before being analyzed by an online mass spectrometer. Sampling is performed only after steady state is reached, typically about 30%minutes after any change in operating conditions. Here, steady state is defined as less than 1%% variation in voltage and outlet concentrations for at least 10%minutes, ensuring all samples reflect the same electrochemical context. Because this is an online analysis, we wait for the mass spectrometer

signal to stabilize—usually about 4%minutes per point—so a full cycle is completed in under half an hour. A schematic system diagram is depicted in **Figure S4**.

The cell-was fed with dry CO_2 on the cathode side, while T = 60 °C 0.05 M $CsHCO_3$ was supplied to the anode (20 cm^3 cm^{-2} min^{-1} normalized feed rate). The CO_2 feed rate was controlled with a Bronkhorst F-201C type mass-flow controller, while an Agilent ADM G6691A type flow meter was used to measure the flow-rate of the cell gas outlet. The pressure of the CO_2 in the electrolyzer was controlled by a FESTO back pressure regulator and measured using an analogue pressure gauge. The electrochemical measurements were performed using a TDK Lambda type power supply, driven by a custom developed LabVIEW software. The measurements were conducted in a two-electrode setup, and the cell voltage is given as the voltage difference between the anode and the cathode.

The composition of the product stream shown in **Figure 2** and **4** were recorded using an Online Infrared Syngas Analyzer, Gasboard-3100 from Cubic Instruments. All concentration profiles were analysed using an UGA300 mass spectrometer from Stanford Research. Its calibration was carried out periodically, using calibrated gas mixtures. The calibration fittings are shown below:

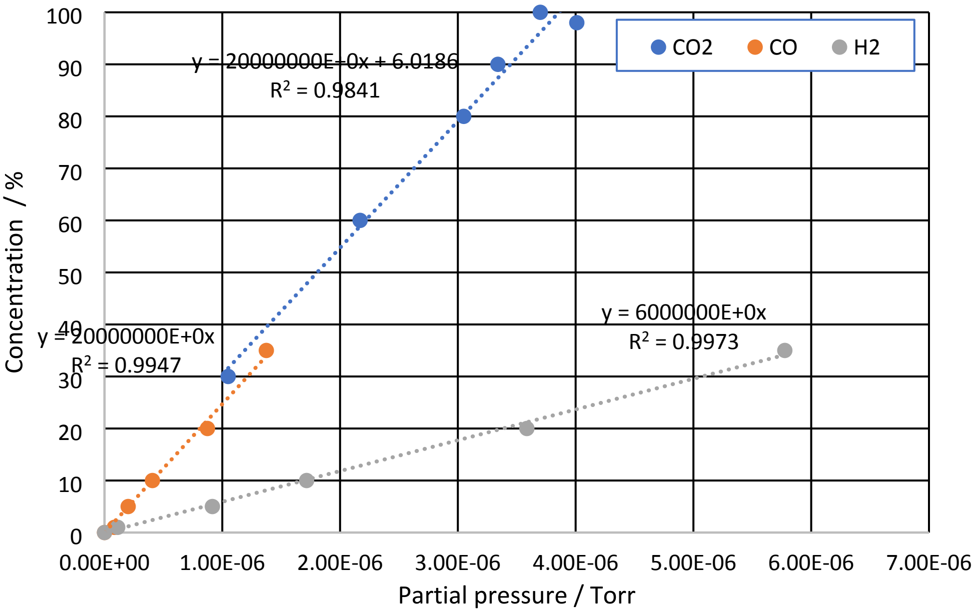

Figure S4. Calibration fittings of the mass spectrometer. The mass spectrometer was periodically calibrated with several gas cylinders, composed of a mixture of CO_2, CO and H_2, in varying ratios.

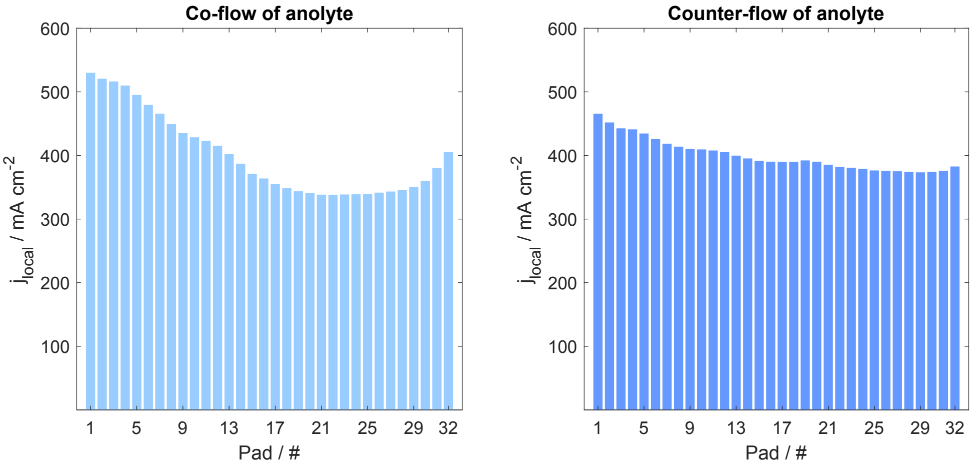

Figure S5. Calibration fittings of the mass spectrometer. The mass spectrometer was periodically calibrated with several gas cylinders, composed of a mixture of CO_2, CO and H_2, in varying ratios.

In the same assembly, and following the same operation conditions as described earlier (this is to say, 0.05 M $CsHCO_3$, 60ºC anolyte and gas, 12 cm^3 cm^{-2} min^{-1} of dry CO_2, 20 cm^3 cm^{-2} min^{-1} of anolyte flow, same MEA preparation as described earlier) we performed 1 hour current controlled (400 mA cm^{-2}) electrolysis, with anolyte flowing co-currently, followed by switching off of the cell, changing the anolyte direction, and 1 hour current controlled (400 mA cm^{-2})

electrolysis, with anolyte flowing counter-currently. Upon stabilization of the current density profiles, we captured the above figures.

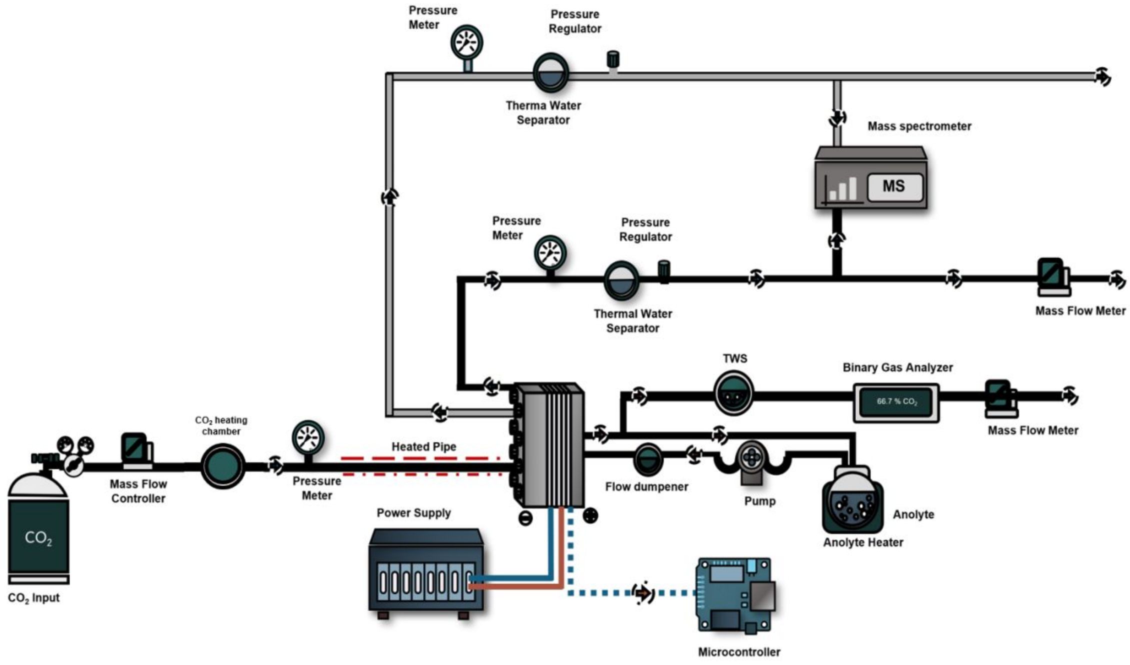

Figure S6. CO_2 electrolyzer system set-up.

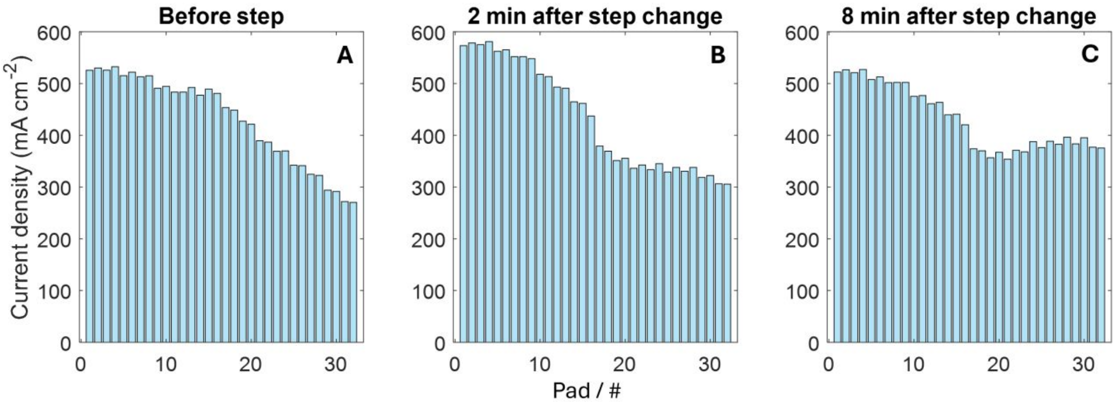

Figure S7. Current density profile evolution when changing CO_2 feed flowrate from 10 cm^3 min^{-1} cm^{-2} to 8 cm^3 min^{-1} cm^{-2}. The nominal average current density was set to 400 mA cm^{-2}. Panel A shows the profile just before the step change, while Panel B and Panel C show the profile 2, and 8 minutes after the step change, respectively.

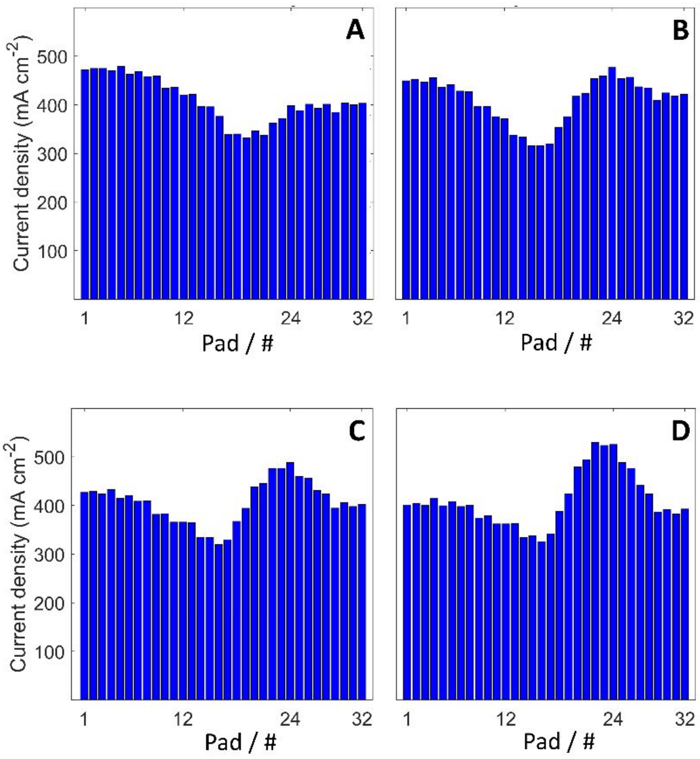

Figure S8. Current density profile evolution when changing CO_2 feed flowrate from 8 cm^3 min^{-1} cm^{-2} to 6 cm^3 min^{-1} cm^{-2}. The nominal average current density was set to 400 mA cm^{-2}. Panel A shows the profile before the change, while Panels B, C, and D show the transition stages, 2, 4 and 12 minutes after the step change in flowrate, respectively.

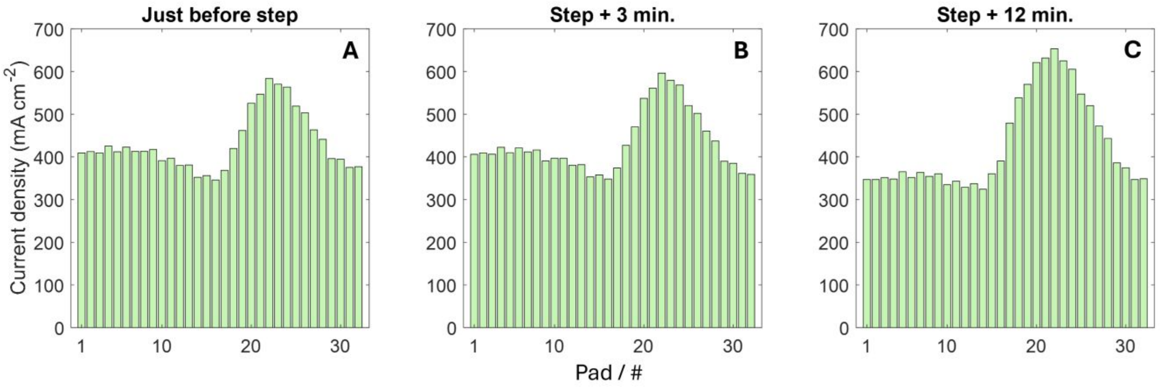

Figure S9. Current density profile evolution when changing CO_2 feed flowrate from 6 cm^3 min^{-1} cm^{-2} to 4 cm^3 min^{-1} cm^{-2}. The nominal average current density was set to 400 mA cm^{-2}. Panel A shows the profile just before the step change, while Panel B and Panel C show the profile 3, and 12 minutes after the step change, respectively.

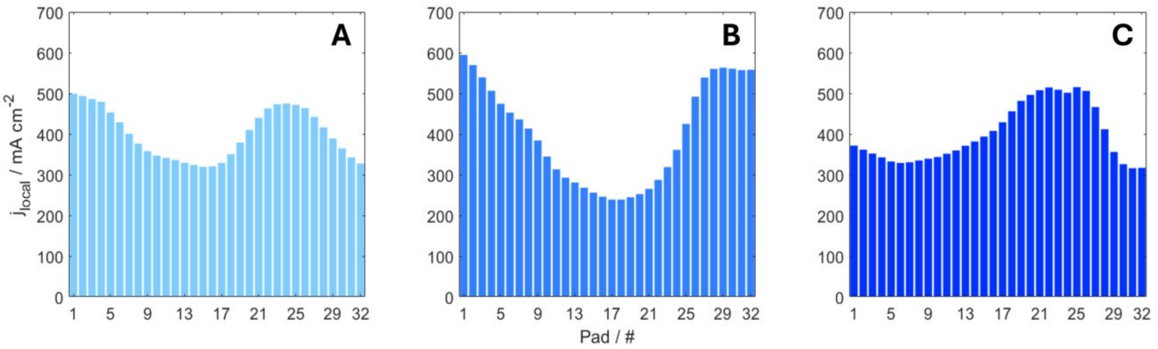

Figure S10. Current density profile after 15 min. of changing CO_2 feed flowrate for (**A**) 8, (**B**) 6 and (**C**) 4 cm^3 min^{-1} cm^{-2}. The nominal average current density was set to 400 mA cm^{-2}.

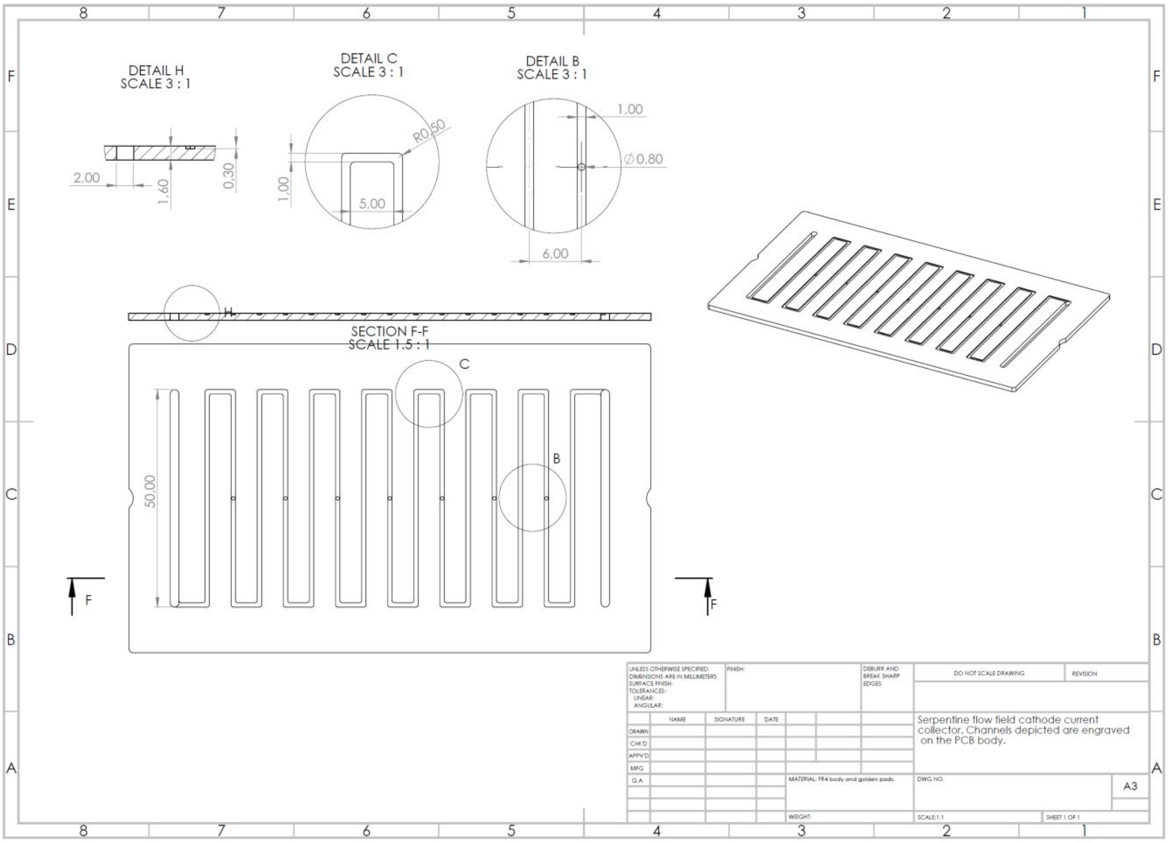

Figure S11. Technical schematic of the serpentine flow field used. The design of the flow field is devised to keep the measuring capabilities of the cathode collector segments. The transversal channels of the flow field were carved through the non-electrically conductive region, which are perpendicular to the flow direction. In this way, the flow field effect can be studied without substantially compromising the conductive nature of the segments. The serpentine flow field is integrated in the PCB body, as the original current collector model was modified through CNC machining to include the flow field. Please note the printed electronics are removed from this CAD drawing, which intends to focus on the flow field itself, thus strictly concerned with the cathode current collector part of the PCB (that that is in contact with CO_2, GDE or sealing).

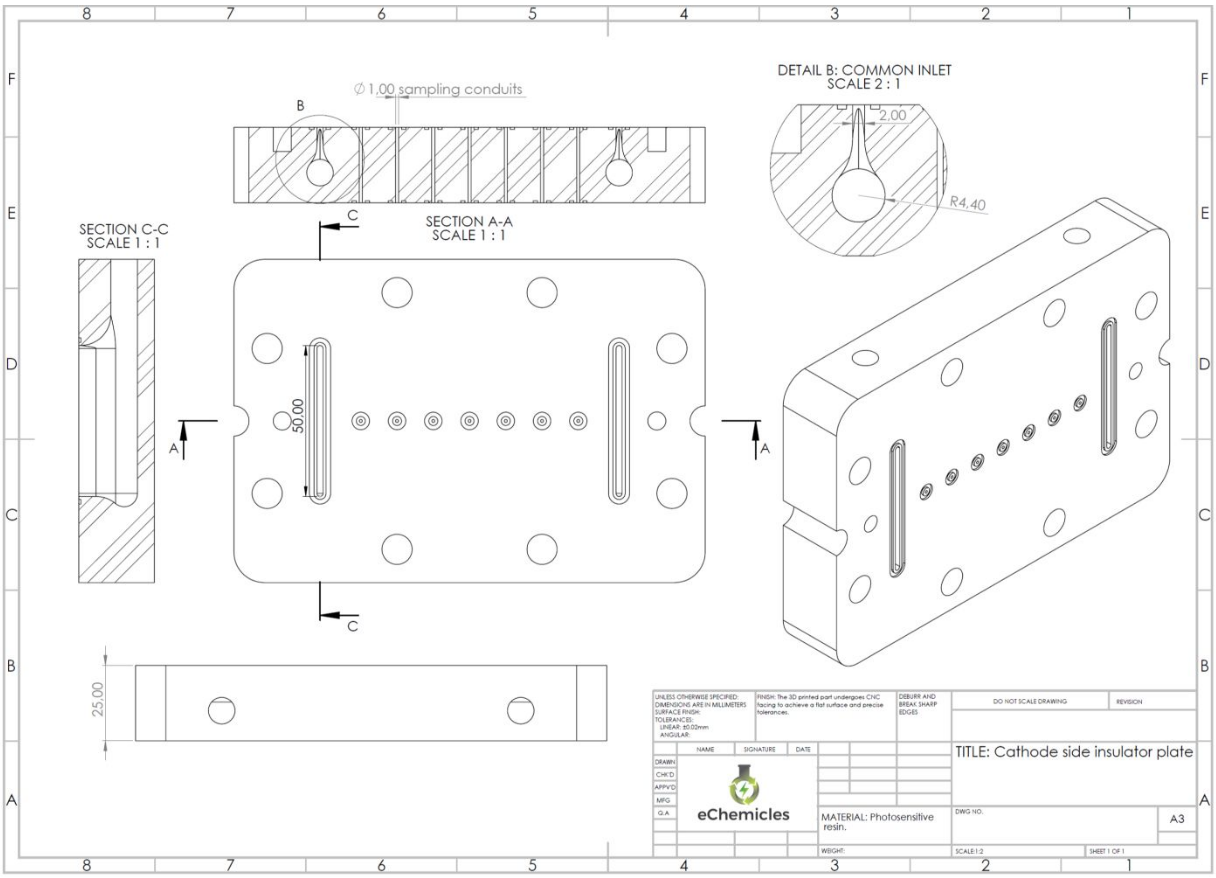

Figure S12. 3D printed insulator plate contains the external inlet and outlet cathode ports of the cells, contains the sampling conduits (Section A-A), and serves as electrical insulator. CO_2 enters through a full-width slot in the insulator plate between the PCB and endplate and exits similarly on the opposite side. To ensure uniform distribution before reaching the active area, the plate includes two buffer chambers—one at the inlet and one at the outlet—that equalize pressure and spread CO_2 evenly across the 5 cm active width. A single small port would not achieve this, hence the slot design.

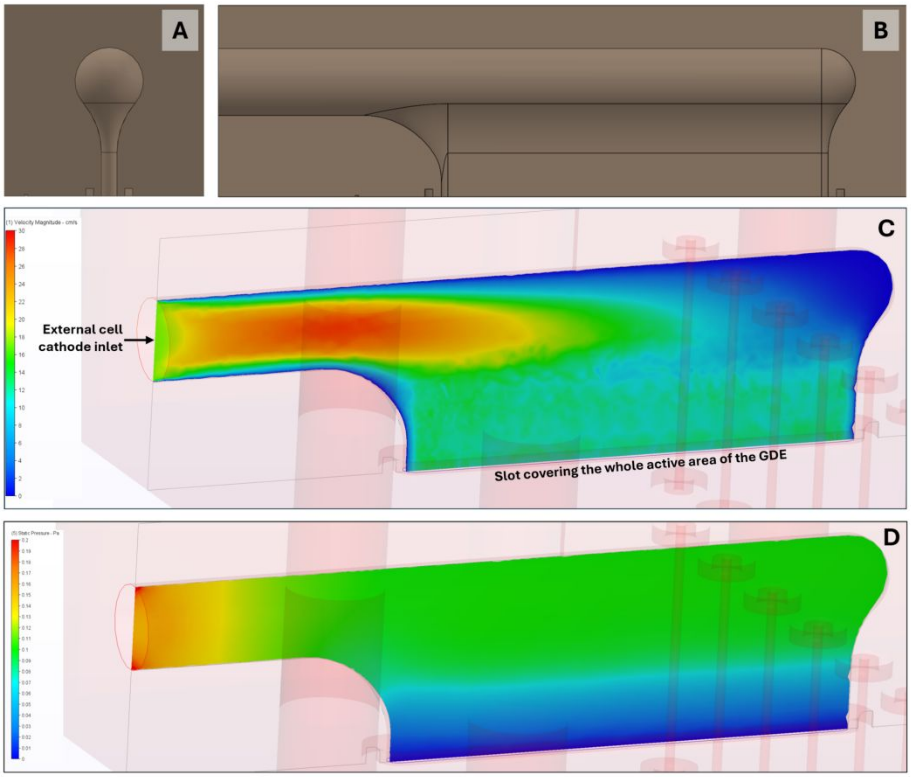

Figure S13. Representation of the simulation of the inlet buffer chamber. CO_2 is fed from the external cell cathode inlet (Boundary: input 600 mL min^{-1} of CO_2, the equivalent of 12 mL min^{-1} cm^{-2}) and exits the chamber through the slot (Boundary: Pa(g) is 0). Further details on the simulation methodology are included below. showing the appropriate dispersion and pressure equalization through the entire width of the GDL, by assessing an even velocity and pressure magnitude at the slot in contact with the GDL. **A.** Section view of the buffer chamber, equivalent to Detail B in **Figure S12**. **B.** Transversal view of the buffer chamber, equivalent to Section C-C in **Figure S12**. **C.** Mid-plane of the chamber and slot showing the even velocity distribution. **D.** Mid-plane of the chamber and slot showing the utility of the chamber in equalizing static pressure in Pa(g). The boundary conditions were set at the external inlet and outlet of the insulator plate (see figure above). Inlet flowrate was set at 600 mL min^{-1}. Outlet flowrate and pressure were set at 600 mL min^{-1} and 0 barg, respectively. CO_2 was modelled as an ideal compressible at 60 ^{o}C (Density, compressibility and others are tailored by the software for CO_2) and solved through a k-epsilon turbulence model.

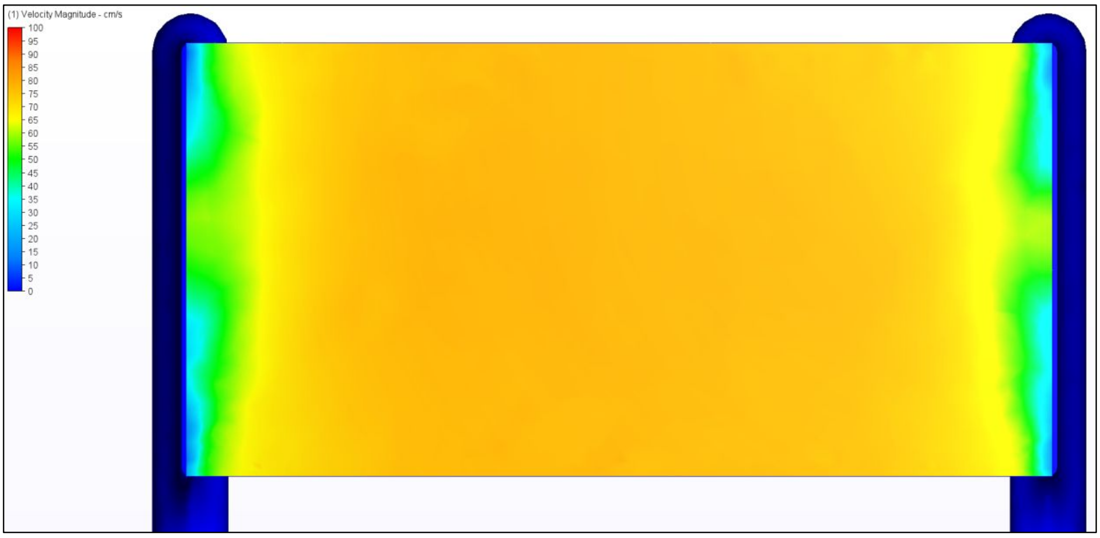

Figure S14. XY-plane mid-section result of the GDE simulation, supporting the idea that such an assembly provides an adequate feeding of the gas to the cell, as proven by a consistent velocity magnitude of ~75 cm/s. All electrochemically induced phenomena (reactions, heating, water, or CO_2 migration, etc.) are excluded from the simulation, for the sake of focusing solely on the geometry effects. The GDE was modelled as a 300 μm x 50 mm x 100 mm homogeneous porous medium. The porous medium is surrounded by its gasket and sandwiched by the cathode current collector/flow field and membrane (adapted for ease of simulation, material set to silicon). CO_2 is fed from the external cell cathode and recovered on the other external port of the insulator plate (Inlet boundary condition: 600 mL min^{-1} of CO_2, the equivalent of 12 mL min^{-1} cm^{-2}; outlet boundary condition: 0 bar (g)). Momentum loss through the GDE is modelled using a Darcy-type resistance (appropriate for porous media) with a fixed through-plane permeability of 10^{-12} m^2 as per the GDL provider information. The simulations were performed in CFD 2024 (Autodesk). In both cases, pure CO_2 was used as the modelled fluid.
